# A meta-analysis of the efficacy and safety of trofinetide in patients with rett syndrome

**DOI:** 10.1007/s10072-024-07584-8

**Published:** 2024-05-21

**Authors:** Mohamed Abo Zeid, Amr Elrosasy, Rashad G. Mohamed, Alina Ghazou, Elarbi Goufa, Nourhan Hassan, Yasmine Abuzaid

**Affiliations:** 1 Faculty of Medicine, Tanta University, Tanta, Egypt; 2 Faculty of Medicine, Cairo University, Cairo, Egypt; 3 Mansoura Manchester Program for Medical Education, Faculty of Medicine, Mansoura University, Mansoura, Egypt; 4 Faculty of Medicine, Jordan University of Science and Technology, Ar-Ramtha, Jordan; 5 Faculty of Medicine, University of Oran 1 - Ahmed Ben Bella, Es Senia, Algeria; 6 Faculty of Medicine, Zagazig University, Zagazig, Egypt

**Keywords:** Rett syndrome, Neurodevelopmental disorder, Genetic disorder, Trofientide, Quality of life

## Abstract

**Background:**

Rett syndrome (RTT) is an uncommon inherited neurodevelopmental disorder that affects brain development, mostly in females. It results from mutation in MECP2 gene in the long arm (q) of the X chromosome.

**Objective:**

Trofinetide is a recently developed drug that has a neuroprotective effect on neurons, and it is our aim in this meta-analysis to evaluate its efficacy and safety in treating Rett syndrome patients.

**Methods:**

We searched 5 databases (PubMed, Scopus, Embase, Web of Science, and Cochrane Library databases) to identify randomized controlled trials (RCTs) comparing Trofinetide and placebo in patients with Rett syndrome until August 13, 2023.Our primary outcomes were the Clinical Global Impression-Improvement (CGI) and the Rett syndrome Behavior Questionnaire (RSBQ). We used Risk of Bias Assessment tool-2 (ROB2) to assess the methodological quality of the included randomized controlled trials.

**Results:**

Three RCTs with a total of 325 patients were included with a follow-up duration ranging from one month to three months. 186 patients received the intervention drug (Trofinetide) and 138 received the placebo. Trofinetide was found to reduce CGI and RSBQ significantly more than placebo (MD = -0.35, 95% CI [-0.52 to -0.18], P 0.0001), (MD = -3.40, 95% CI [-3.69 to -3.12], P 0.00001) respectively. Most adverse events did not show any statistical difference between Trofinetide and the placebo.

**Conclusion:**

Trofinetide offers promise as a potential effective and safe therapeutic opportunity for a population without many available treatments, with improvements seen on both CGI and RSBQ assessments and no severe adverse effects reported.

## Introduction

Rett syndrome (RTT) is an x-linked neurodevelopmental disease that primarily affects females, with an incidence between 1:10,000 and 1:15000 [1]. RTT develops due to a gene mutation on the long arm (q) of the X chromosome. Methyl CpG-binding protein 2 (MECP2) is the protein that the gene encodes. The primary role of MECP2 is the activation and suppression of transcription, which is crucial for the maturation of neurons and their functions. Therefore, suppression of the gene leads to wide disruption of the activity of many other genes and defects the epigenetic regulatory molecules [2–4].

RTT syndrome presents itself with different variants. In the first six months of life, the classic RTT starts with normal development, which is followed by years of development regression. The symptoms are classified into four stages: stagnation (age 6–18 months), rapid regression (age 1–4 years), pseudo-stationary (age 2–potentially life), and late motor deterioration (age 10–life). [4–6]. Patients present in different ways, which results in a variety of neuromotor and cognitive disabilities. Symptoms begin with some autistic behaviors such as complete or partial loss of expressive language, failure to maintain eye contact in addition to loss of purposeful hand movement and impaired gait [7–9]. Recently there is evidence that atypical behaviors could start to appear before the regression begins, indicating that the syndrome is not entirely symptom-free in the first few months of life. [10, 11]

Until recent years treatment of RTT depended mainly on controlling the symptoms. Management of all the disabilities requires a multidisciplinary approach which varies from physiotherapy of the affected muscles to controlling some neurological symptoms such as seizures [12].

The advance in neurological research to improve cognitive function has made new discoveries in a lot of neurological disorders. Trofinetide (glycyl-L-2-methylpropyl-L-glutamic acid) is a synthetic analog of glycine–proline–glutamate (GPE), a natural tripeptide in the brain that has a neuroprotective effect on the neurons. In mouse trials, both Trofinetide and GPE improve respiratory and cardiac functions as well as increase brain weight in MECP2 deficient mice [13]. These observations led to the conduct of new randomized trials to investigate Trofinetide's impact on RTT patients.

This study's objective is to review that database and assess the efficacy and negative effects of Trofinetide on RTT patients.

## Methods

### Study registration

This meta-analysis followed the guidelines of the PRISMA [14] (Preferred Reporting Items for Systematic Reviews and Meta-Analyses) statement and was registered in the International Prospective Register of Systematic Reviews (Prospero) (registration number: CRD42023455486).

### Criteria for inclusion and exclusion

Our inclusion criteria were based on the PICO acronym as we included only patients with Rett syndrome taking the intervention (Trofinetide) comparing it to placebo. Our primary outcomes are Rett Syndrome Behavior Questionnaire and Clinical Global Impression-Improvement (CGI) while the secondary outcomes are any adverse events and we included only randomized controlled trials. We excluded: Any study designs other than RCTs, conference abstracts, case reports and case series, systematic reviews, meta-analyses, and studies with full text unavailable.

### Search strategy and selection process

PubMed, Scopus, Midline, Embase, Web of science, Cochrane Library databases were systematically searched for relevant randomized Controlled Trials until 13th August. The terms used to develop the search strategy were "Trofinetide," "Rett Syndrome," and " Rett " and we used Boolean logic “AND” to connect terms. We limited the studies language to English, and subjects to human. Two independent reviewers blindly screened All titles and abstracts determined by the primary search according to our pre-specified exclusion and inclusion criteria using Rayyan website [15]. After that the full text was screened for final inclusions. A third reviewer was involved to resolve any discrepancies.

### Data extraction

Two authors independently conducted the data extraction with the help of an online data sheet. The data that were extracted comprised the following information: characteristics of the studies that were included and their populations, risk of bias categories, and outcome measures. Any conflicts in screening or data extraction were resolved via discussion with a third author.

### Quality assessment

Version 2 of the Cochrane risk-of-bias tool for randomized trials (RoB-2) [16] was used to assess the methodological quality of the included randomized controlled trials. The RoB was evaluated according to the following five domains: (1) bias in the randomization process; (2) bias arising from the deviations from the intended interventions; (3) bias arising due to lost outcome data; (4) bias in the outcome's measurement; (5) bias in the reported result's selection. Each RCT was categorized as being a high, low, or unclear risk for the above-mentioned domains. Each study was independently assessed by two authors, and any discrepancies were resolved via discussion with a third author.

### Data synthesis

Review Manager (RevMan) 5.4 software will be used to conduct the meta-analysis [17]. Fixed effect model will be used in case of absence of heterogeneity and random effect model in case of heterogeneity qualitative analysis will also be conducted using tables and figures.

To measure the effect of the dichotomous outcomes (Adverse events (AEs), serious AEs, diarrhea, vomiting, pyrexia, seizures, and irritability) the risk ratio (RR) was used, while for continuous variables (Change in MBA from baseline to end of follow up and Change in RSBQ from baseline to the end of follow up) the mean difference (MD) was used. For each outcome of interest, a set point estimate with a 95% confidence interval (CI) was used, and a *p* < 0.05 was considered statistically significant.

### Assessment of heterogeneity

The Chi-Square (χ2) and I2 tests were used to assess heterogeneity. The χ2 test was used to determine the presence of considerable heterogeneity, with p ≤ 0.10 recognized as statistically significant. The I2 test was used to measure heterogeneity. The heterogeneity was classified as not significant (30%), moderate (30–50%), large (51–75%), and significant (76–100%).

## Results

### Search results and study selection

**Fig. 1:**
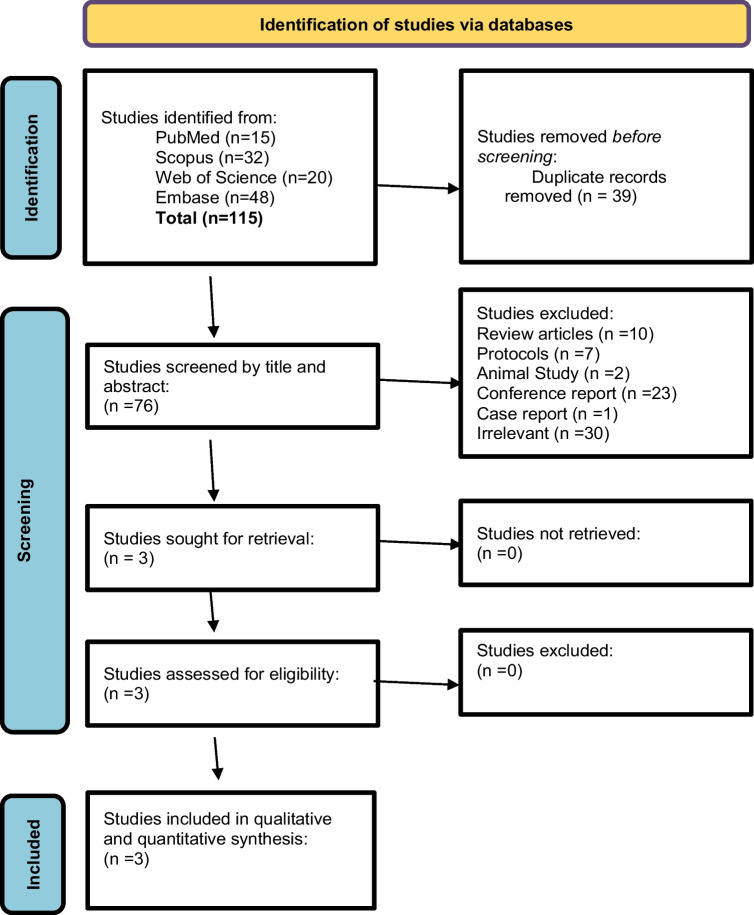
PRISMA flow diagram of the included studies

On searching the different electronic databases (PubMed, Scopus, Embase and Web of science) using the predefined search strategy, a total of 115 studies were initially retrieved, 39 of them were found to be duplicated and were removed. During the title and abstract screening and with applying the inclusion and exclusion criteria mentioned above on the remaining 76 studies, 73 studies were found to be irrelevant. As a result, three full‐text articles [18–20] were evaluated for eligibility and were found to be relevant to our topic (Fig. 1).

### Characteristics of included studies

We included a total of three studies (RCTs) [18–20] including 325 patients with a follow-up duration ranging from one month to three months. The three studies were carried out in the USA using different doses of the drug ranging from 35 mg/kg to 500 mg/kg (Table 1).

**Table 1:** summary of the included studies

Study ID	Study Design	Country	Number of centers	Total participants	Follow-up duration	Main inclusion criteria	Primary Outcomes	Conclusion
Glaze 2017	Double-blinded phase 2 RCT	USA	3	56	28 days	adolescent and adult females with Rett syndrome	1. Safety assessments included adverse events, clinical laboratory tests, vital signs, electrocardiograms, physical examinations, and concomitant medications 2. Efficacy measurements were categorized into 4 efficacy domains, which related to clinically relevant, phenotypic dimensions of impairment associated with Rett syndrome	1. Both 35 mg/kg and 70 mg/kg dose levels of Trofinetide were well tolerated and generally safe" 2. In comparison to placebo, Trofinetide at 70 mg/kg showed efficacy according to established criteria. At the increased dose, multiple outcome measures that evaluate crucial aspects of Rett syndrome showed continuous effectiveness trends
Glaze 2019	Double-blinded phase 2 RCT	USA	12	82	66 days	1. classic/typical Rett syndrome with MeCP2 gene mutation 2. Age 5—15 years 3. Weight between 15.0- 100.0 kg at Screening and Baseline. 4.participant can ingest the drug administered orally or through a gastrostomy tube.	1. Safety assessments: Adverse events 2. Motor Behavior Assessment Scale (MBA) 3. Clinical Global Impression of Improvement (CGI-I) 4. Caregiver Top 3 Concerns via a Visual Analogue Scale (VAS)	1. All dose levels were well tolerated and generally safe 2. In comparison to placebo, rofinetide at 200 mg/kg bid showed statistically significant and clinically relevant improvements on the Rett Syndrome Behavior Questionnaire, RTT-Clinician Domain Specific Concerns–Visual Analog Scale, and Clinical Global Impression Scale
Neul 2023	Double-blinded phase 3 RCT	USA	21	187	12 weeks	1. Female patients 5 to 20 years at Screening 2. Body weight ≥ 12 kg at Screening 3. Able to swallow the study drug provided as a liquid solution or through gastrostomy tube 4. classic/typical Rett syndrome 5. An established MECP2 gene mutation that causes disease 6. Has a stable seizure pattern or has not experienced any seizures within eight weeks of the screening 7. participants in reproductive period must agree to utilize legal methods of contraception or refrain from sexual activity for the duration of the study and for at least 30 days after & Subject must not be pregnant or breastfeeding. 8. The participant 's caregiver is English-speaking and can complete the caregiver assessments 9. participant and caregiver(s) must live in a place where the study drug can be supplied and have lived there for at least three months before screening	1. The change from baseline to week 12 in RSBQ total score 2. The change from baseline to week 12 in CGI-I scale score at week 12 3. The change from baseline to week 12 in the CSBS-DP-IT Social Composite score 4. A prespecified subgroup analysis examined treatment effects by age, baseline RSBQ severity and MECP2 mutation severity as categorized according to the RTT Natural History Study41	The coprimary efficacy endpoints showed a significant improvement for Trofinetide when compared to placebo, indicating that Trofinetide is effective in treating the main Rett syndrome symptoms

RCT, randomized controlled trial; RTT, MBAS, Motor Behaviour Assessment Scale; CGI-I, Clinical Global Impression of Improvement; VAS, Visual Analogue Scale; RSBQ, Rett Syndrome Behaviour Questionnaire; CSBS-DP-IT, the Communication and Symbolic Behavior Scales Developmental Profile Infant–Toddler Checklist; BID, twice a day

Of these, 186 patients received the intervention drug (Trofinetide) and 138 received the placebo. The average age was 13.1 years in the intervention group and 13.3 years in the control group (Table 2)**.**

**Table 2:** Baseline characteristics of enrolled patients in the included studies

Study ID	Study groups	Age (Years) Mean (SD)		Weight mean (SD)		Height mean (SD)		Race n (%)			
								White		Black	
		I	C	I	C	I	C	I	C	I	C
Glaze 2017	**cohort 0 (35 mg/kg for 14 day)**	26.65 (8.78)	22.43 (4.61)	NA	NA	NA	NA	5 (100)	3 (75)	0	1 (25)
	**cohort 1 (35 mg/kg for 28 day)**	22.62 (5.58)	32.09 (9.32)	NA	NA	NA	NA	10 (77)	5 (100)	3 (23)	0
	**cohort 2 (70 mg/kg for 28 day)**	24.52 (5.85)	27.09 (8.36)	NA	NA	NA	NA	15 (88)	11 (100)	1 (6)	0
Glaze 2019	**50 mg/kg**	10.06 (3.18)	9.38 (3.26)	26.13 (9.78)	24.20 (6.87)	124.12 (11.70)	122.69 (12.67)	15 (100)	22 (92)	0	0 (0)
	**100 mg/kg**	10.81 (3.10)	32.09 (9.32)	30.43 (12.16)	NA	129.55 (12.76)	NA	15 (94)	5 (100)	1 (6)	0
	**200 mg/kg**	9.23 (3.88)	27.09 (8.36)	25.22 (11.51)	NA	121.55 (15.19)	NA	25 (93)	11 (100)	0	0
Neul 2023		11.0 (4.69)	10.9 (4.57)	NA	NA	NA	NA	82 (88.2)	90 (95.7)	1 (1.1)	1 (1.1)

Study ID	Race n (%)				Ethnicity n (%)				BMI mean (SD)		
	Asian		Other		Hispanic		Non-hispanic				
	I	C	I	C	I	C	I	C	I	*C*	
Glaze 2017	0	0	0	0	0	2 (50)	5(100)	2 (50)	19.27 (3.139)	23.96 (1.81)	
	0	0	0	0	0	1 (20)	13 (100)	4 (80)	25.06 (7.930)	24.66 (8.04) kg/ m^2^	
	1 (6)	0	0	0	2 (12)	0	15 (88)	11 (100)	20.48 (6.765)	19.24 (3.6) kg/ m^2^	
Glaze 2019	0	1 (4)	0	1 (4)	1 (7)	0 (0)	14 (93)	24 (100)	16.50 (3.61)	16.00 (2.85)	
	0	0	0	0	1 (6)	1 (20)	14 (88)	4 (80)	17.70 (5.06)	16.00 (2.85)	
	2 (7)	0	0	0	6 (22)	0	21 (78)	11 (100)	16.31 (3.57)	16.00 (2.85)	
Neul 2023	5 (5.4)	1 (1.1)	2.5 (3.5)	2 (2.1)	NA	NA	NA	NA	NA	NA	

NA, not available; SD, standard deviation, n, number; BMI, body mass index

### Risk of bias and quality of evidence

**Fig. 2:**
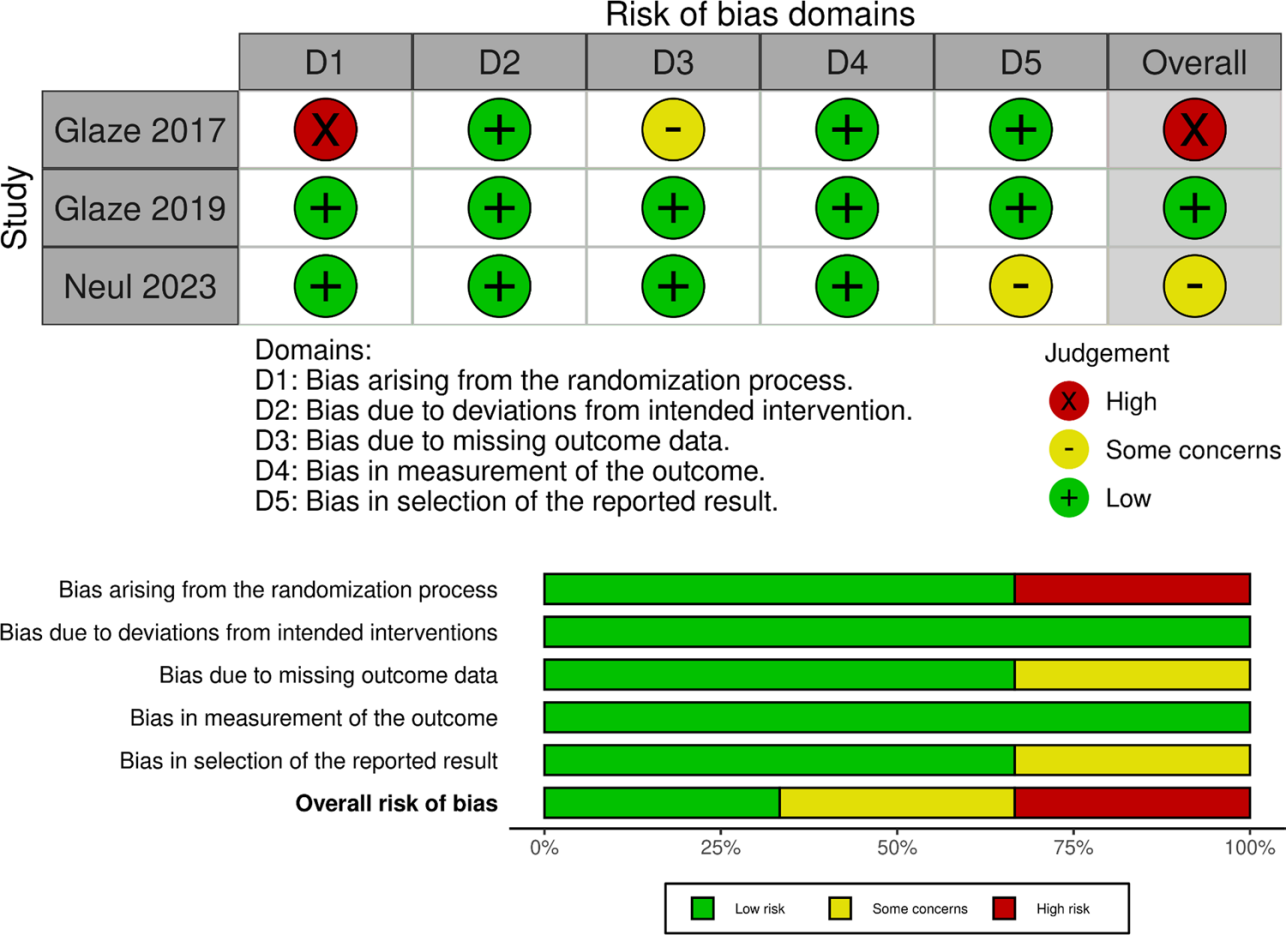
Summary of assessment of risk of bias

We used the tool Cochrane RoB2 with five domains to evaluate each outcome included in the quantitative synthesis’s risk of bias. One study [18] showed a high risk of bias mainly in the randomization process and the missing outcome data domains, another one [19] showed a low risk of bias, while the third one [20] showed some concerns mainly in the fifth domain (Selection of the reported result) (Fig. 2).

### Efficacy outcomes

Glaze 2017 used two doses (35 mg and 70 mg), while Glaze 2019 used three doses of the drug (50, 100, 200 mg) and Neul 2023 used different doses ranging from (200 to 500 mg). Our analysis found that Trofinetide scored higher than the placebo on the CGI and RSBQ scales. However, it was also found to be less safe than the placebo, particularly in terms of causing diarrhea and vomiting.

#### Clinical Global impression improvement (CGI)

**Fig. 3:**
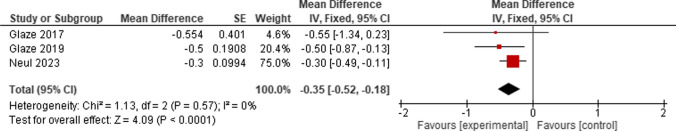
Forest plot for Clinical Global Impression-Improvement (CGI)

Comparing the Trofinetide group to the control group the total mean difference between them favored the Trofinetide group. (MD = -0.35, 95% CI [-0.52 to -0.18], *p* < 0.0001), with no heterogeneity among the included studies (*P *= 0.57, I-square = 0%) (Fig. 3).

#### Motor Behavior Assessment Scale (MBA)

**Fig. 4:**
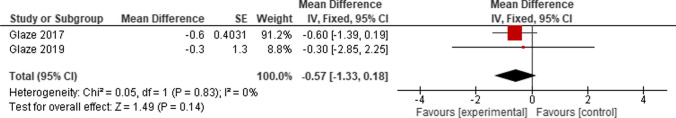
Forest plot for Motor Behavior Assessment Scale (MBA)

When comparing the Trofinetide group to the control group the total mean difference between them did not favor either of them. (MD = -0.57, 95% CI [-1.33 to 0.18],* P* = 0.14), with no heterogeneity among the included studies (*P* = 0.83, I-square = 0%) (Fig. 4).

#### Rett Syndrome Behavior Questionnaire (RSBQ)

**Fig. 5:**
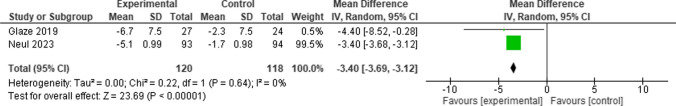
Forest plot for Rett Syndrome Behavior Questionnaire (RSBQ)

Comparing the Trofinetide group to the control group the total mean difference between them was in favor of the Trofinetide group. (MD = -3.40, 95% CI [-3.69 to -3.12], *p* < 0.00001), with no heterogeneity among the pooled studies (*P* = 0.64, I-square = 0%) (Fig. 5).

### Safety outcomes

#### Adverse events

**Fig. 6:**
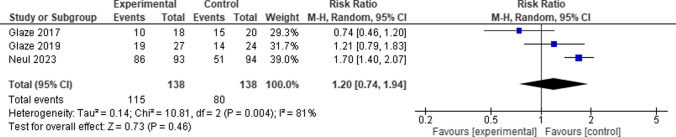
Forest plot for Adverse events

When comparing the Trofinetide group to the control group, the overall risk ratio between them did not favor either of them. (RR = 1.20, 95% CI [0.74 to 1.94], *P* = 0.46), and the pooled studies showed significant heterogeneity among them (*P* = 0.004, I-square = 81%) (Fig. 6).

#### Diarrhea

**Fig. 7:**
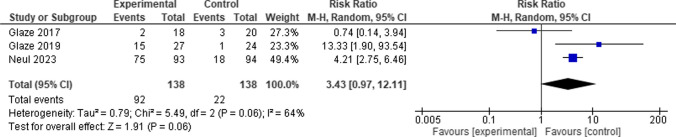
Forest plot for Diarrhea

When comparing the Trofinetide group to the control group, the overall risk ratio between them favored the control group. (RR = 3.43, 95% CI [0.97 to 12.11], *P* = 0.06), and the pooled studies showed large heterogeneity among them (*P* = 0.06, I-square = 64%) (Fig. 7).

#### Vomiting

**Fig. 8:**
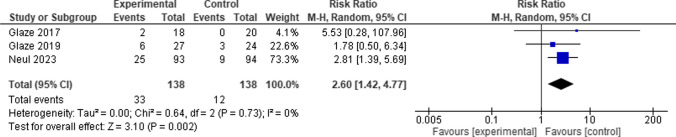
Forest plot for Vomiting

When comparing the Trofinetide group to the control group, the overall risk ratio between them favored the control group. (RR = 2.60, 95% CI [1.42 to 4.77], P = 0.002), and the pooled studies revealed no heterogeneity among them (P = 0.73, I-square = 0%) (Fig. 8).

#### Pyrexia

**Fig. 9:**
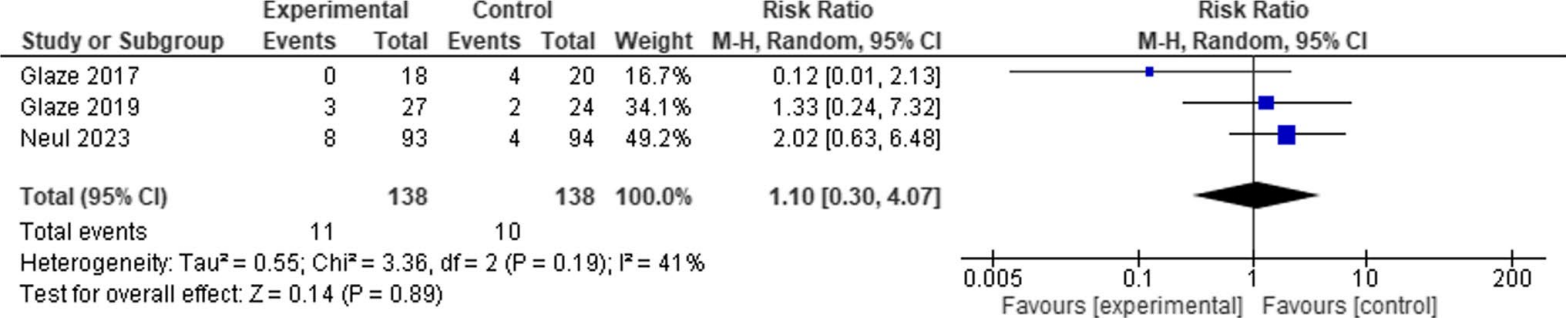
Forest plot for Pyrexia

Comparing the Trofinetide group to the control group, the total risk ratio between them did not favor either of the two groups. (RR = 1.10, 95% CI [0.30 to 4.07], *P* = 0.89), and the pooled studies showed moderate heterogeneity among them (*P* = 0.19, I-square = 41%) (Fig. 9).

#### Seizures

**Fig. 10:**
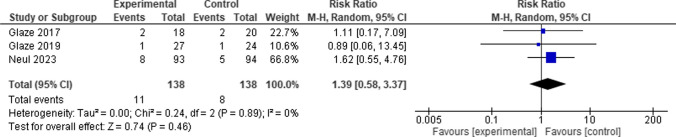
Forest plot for Seizures

Comparing the Trofinetide group to the control group, the total risk ratio between them was not in the favor of either of them. (RR = 1.39, 95% CI [0.58 to 3.37], *P* = 0.46), and the pooled studies revealed no heterogeneity among them (*P* = 0.89, I-square = 0%) (Fig. 10).

#### Serious adverse events

**Fig. 11:**
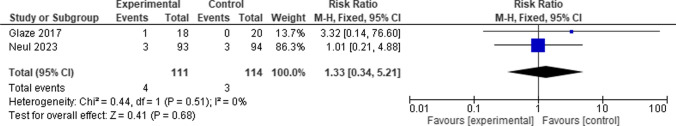
Forest plot for Serious adverse events

Comparing the Trofinetide group to the control group the total risk ratio between them was not in favor of either of them. (RR = 1.33, 95% CI [0.34 to 5.21], *P* = 0.68), and the pooled studies revealed no heterogeneity among them (*P* = 0.51, I-square = 0%) (Fig. 11).

## Discussion 

Three RCTs with 325 RTT patients were included comparing Trofinetide to placebo showing statistically significant difference on the CGI implying that Trofinetide has a positive effect on the clinical condition of RTT patients. Trofinetide, also achieved better RSBQ scores compared to placebo, however and according to Hou 2020 [21] the RSBQ has its limitations and potential problems when applied in clinical trials for RTT, so it would be unwise to completely rely on RSBQ to determine the efficacy of the drug. Based on these results Trofinetide could target the key symptoms of RTT such as impaired communication, compulsive behaviors, and emotional disorders, thus improving the lives of patients and their families.

Several adverse events were analyzed such as diarrhea, vomiting, fever, seizure, and irritability. There were no clear significant differences in the total rates of adverse events between Trofinetide and the placebo groups: [Neul et al., 2023] [20]. However, most AEs were low or moderate-grade and managed with appropriate interventions [Neul et al., 2023] [20]. When evaluating the gastrointestinal issues that mattered in this evaluation, including diarrhea and vomiting. Diarrhea was more common with Trofinetide but was typically transient, resolving on drug cessation, while vomiting tended to be more frequent in the control group suggesting that subjects on placebo have a poorer tolerance [Neul et al., 2023] [20]. No significant difference was found between Trofinetide and placebo for pyrexia (fever), seizures, and irritability indicating that Trofinetide did not aggravate those symptoms in RTT patients.

A meta-analysis of three RCTs on Trofinetide in RTT patients revealed statistically significant improvements in CGI and RSBQ scores, suggesting potential clinical benefits. Safety analysis showed minor adverse events with relatively low risk. This comprehensive review provides an overall view of Trofinetide's effectiveness and safety in treating RTT symptoms, indicating its promise as a treatment option.

The incorporated RCTs showed limited lengths of 1–3 months of post-discharge follow-up. It constrained our capacity to evaluate the durability of security and viability of Trofinetide with RTT sufferers over the long haul. Also. some safety outcomes, including adverse events, showed heterogeneity in our analysis, so these results cannot be generalized to the whole population of patients with RTT, as such heterogeneity could be attributed to the differences in drug doses, patients’ attributes, or other reasons among the included trials; Glaze 2017 examined mainly adolescents and adults while the other two trials included children; the youngest patient in Glaze 2017 was 15 years old with mean age 25 years, on the other hand, Glaze 2019 examined patients between the ages of 5 and 15, therefore Glaze 2017 and Glaze 2019 are not examining the same patient’s populations. Glaze 2019 performed its final efficacy analysis at day 54, while Glazed 2019 measured its efficacy outcomes at day 66 (almost 9.5 weeks) and Neuls 2023 measured its efficacy at 12 weeks, a follow up duration of approximately twice as long Glaze 2019. Also, the three studies used completely different doses of Trofinetide; Glaze 2017 used two doses (35 mg and 70 mg), while Glaze 2019 used three doses of the drug (50, 100, 200 mg) and Neul 2023 used different doses ranging from (200 to 500 mg). It is also important to note that Glaze 2017 showed high risk of bias by using RoB 2 and Neul 2023 showed some concerns, while Glaze 2019 showed low risk of bias which could also be attributed to the heterogeneity among them. Although, there was some heterogeneity among the three studies it was unapplicable to perform sub-group analysis due to the lack of sufficient number of trials to be included since RTT is a rare disorder and Trofinetide is a new drug that is not widely available. The trial underscores the importance of consistent reporting of safety results from clinical studies in the future.

Long-Term Follow-up: It would be of value for future research to have longer-term outcomes investigating the sustainability of the impact and to offer some information on the long-term safety profile of Trofinetide.

Dose Optimization: Future research on dose optimization is needed to identify the optimal and most tolerable doses of Trofinetide for various ages and types of RTT. This might be used to personalize medication methods to every single person’s needs. Biomarker Development: Work should be done to find biomarkers to determine which Trofinetide-induced physiologic, neurocognitive, or behavioral changes may have occurred. It would help us to uncover the mechanism of action responsible for how it works and give us a better idea as to whether someone is responding well to their treatment. Inclusion of Diverse Populations: Extending the study participants' age range (to children and adolescents with Rett syndrome) will offer a better understanding of Trofinetide effectiveness and safety.

## Conclusion

Overall, the results from our meta-analysis imply that Trofinetide has the potential to be an effective therapy for the classic features of Rett syndrome, nevertheless improvements must be done for future studies regarding the heterogeneity and the follow up duration.

## Data Availability

The datasets used and/or analyzed during the current study are available from the corresponding author on reasonable request.
